# Odevixibat therapy in progressive familial intrahepatic cholestasis with MYO5B variants: a retrospective case series

**DOI:** 10.1186/s13023-025-03728-x

**Published:** 2025-05-12

**Authors:** Bertrand Roquelaure, Marco Sciveres, Tassos Grammatikopoulos, Eberhard Lurz, Folke Freudenberg, Dalila Habes, Lionel Thevathasan, Fatine Elaraki, Emmanuel Gonzales

**Affiliations:** 1 Service de Pédiatrie Multidisciplinaire, APHM, Hôpital de la Timone Enfants, Marseille, France; 2 Pediatric Hepatology and Pediatric Liver Transplantation, ISMETT, University of Pittsburgh Medical Center Italy, Palermo, Italy; 3 Liver Unit and Liver Transplant Program, Bambino Gesù Children Hospital, Rome, Italy; 4 Institute of Liver Studies, King’s College London, London, UK; 5 Paediatric Liver, GI and Nutrition Centre and Mowat Labs, King’s College Hospital NHS Trust, London, UK; 6 Division of Paediatric Gastroenterology and Hepatology, Dr. von Hauner Children’s Hospital, University Hospital Munich, LMU, Munich, Germany; 7 Klinikum Dritter Orden, Division of Pediatric Gastroenterology and Hepatology, Munich, Germany; 8 Hépatologie et Transplantation Hépatique Pédiatriques, Centre de Référence de l’Atrésie des Voies Biliaires et des Cholestases Génétiques, AP-HP, FSMR FILFOIE, ERN RARE LIVER, Hôpital Bicêtre, Université Paris-Saclay, Inserm U 1193, Hépatinov, Paris, France; 9 LT Associates Ltd, Purley, UK; 10 Ipsen, Boulogne, France

**Keywords:** Progressive Familial intrahepatic cholestasis, Myosin 5B, Pruritus, Bilirubin, Odevixibat, Serum bile acid, Ileal bile acid transporter inhibitor

## Abstract

**Background and rationale:**

Progressive familial intrahepatic cholestasis (PFIC) associated with myosin 5B deficiency is a rare liver disease characterised by elevated serum bile acids (sBAs) and severe pruritus. The objective of this study was to evaluate treatment with the ileal bile acid transporter inhibitor odevixibat in affected children.

**Methods:**

This was a retrospective analysis of five children with a diagnosis of PFIC associated with myosin 5B deficiency and pruritus refractory to treatment with rifampicin and ursodeoxycholic acid, starting odevixibat treatment (37.2–120 µg/kg.day) between 15 months and 10 years of age. Clinical and laboratory data were collected regularly, including liver biochemistry and treatment history. Pruritus and sleep disorders were rated on a four-point Likert scale (absent, mild, moderate or severe).

**Results:**

In the year before starting odevixibat, all patients presented with moderate to severe refractory pruritus. Four patients had sleep disturbances. One patient had a history of microvillus inclusion disease and was parenterally fed during his first year of life. In the year prior to initiating odevixibat, sBA levels were > 150 µmol/L and total bilirubin levels were > 25 µmol/L in all patients. Within six months after starting odevixibat, sBA levels normalised to < 10 µmol/L and total bilirubin fell to < 15 µmol/L. Bilirubin and sBA levels remained mostly normal throughout the treatment period (from 22 to 39 months) in four patients. Pruritus and sleep disturbances improved in the first three months and disappeared completely on treatment in four patients. In two patients, compliance and access to treatment were limited, which may explain the fluctuations in treatment response. In one patient, odevixibat treatment was discontinued following an episode of infectious gastroenteritis leading to a rise in sBA and symptom recurrence which did not respond to treatment reinitiation. Digestive tolerability of odevixibat was good; no new or worsening gastrointestinal symptoms were observed in any child.

**Conclusion:**

This case series indicates that treatment with odevixibat is effective in children with myosin 5B-related PFIC and encourages further research into the utility of this medication in rare forms of PFIC.

**Supplementary Information:**

The online version contains supplementary material available at 10.1186/s13023-025-03728-x.

## Introduction

Progressive familial intrahepatic cholestasis (PFIC) is a family of genetic, infantile-onset diseases characterised by impaired bile acid secretion and hepatocellular cholestasis [1, 2]. Molecular genetic studies have shown PFIC to be heterogenous in origin, the three most common forms being PFIC1, PFIC2, and PFIC3. With the onset of high-performance genetic screening methods, several other, much rarer, genetic defects associated with PFIC have been identified [3]. One of these defects corresponds to a biallelic variant in the *MYO5B* gene encoding myosin 5B, which was reported in 2017 from patients with PFIC of unknown origin [4, 5]. This work was inspired by several earlier observations of cholestatic liver disease in patients with microvillus inclusion disease (MVID), a genetic disease also caused by variants in the *MYO5B* gene [6–8].

The incidence of PFIC associated with myosin 5B deficiency is not known, but is likely to be very low, with less than fifty cases described to date worldwide. Cholestatic disease in these patients is thought to result from incorrect trafficking of transporter proteins in hepatocytes, including the bile salt export pump (BSEP), due to missense variants in myosin 5B [9, 10]. Although both PFIC and MVID can be caused by *MYO5B* variants, most PFIC cases described are not associated with MVID, although intestinal symptoms such as diarrhoea are frequent [4, 5, 11–13]. On the other hand, around 50% of MVID cases present with cholestatic liver disease [14]. Initially, PFIC associated with myosin 5B deficiency was classified as PFIC6, but it has recently been renamed PFIC10 [15].

The principal clinical manifestations are neonatal or infantile jaundice, often transient, and severe chronic pruritus [4, 5, 12]. At the biochemical level, PFIC associated with myosin 5B deficiency is characterised by low to normal levels of gamma-glutamyl transferase (GGT) and elevated serum bile acid (sBA) concentrations. Treatments include ursodeoxycholic acid (UDCA) and rifampicin to decrease cholestasis and pruritus. Surgical biliary diversion or liver transplantation (LT) may be required in patients with refractory pruritus or end-stage liver disease [4, 7, 11, 16].

Odevixibat is a small-molecule inhibitor of the ileal bile acid transporter (IBAT) which has been developed for the treatment of PFIC and other cholestatic liver diseases [17–19]. It has been licensed for the treatment of PFIC in Europe and for the treatment of pruritus in patients with PFIC in the USA since July 2021 [17]. It has also been approved for the treatment of cholestatic pruritus in patients with Alagille syndrome in Europe since September 2024, and in the USA since June 2023. The Phase III PEDFIC1 clinical trial and its open-label long-term extension (PEDFIC2) have shown that odevixibat treatment results in reduction of sBA and improvement of pruritus in a significant proportion of children with PFIC [20, 21]. Although the PEDFIC1 study only included patients with PFIC1 or PFIC2, PEDFIC2 was open to patients with all types of PFIC. One patient with PFIC associated with myosin 5B deficiency, whose clinical features have been described previously [4], was included in PEDFIC2. In addition, since odevixibat was licensed in the European Union, children with PFIC (all types) were eligible for treatment within the framework of a compassionate use programme before odevixibat became commercially available. This study describes outcome in five patients with PFIC associated with myosin 5B deficiency treated with odevixibat.

## Methods

### Patients

This was a retrospective study of children with PFIC associated with myosin 5B deficiency, who had started treatment with odevixibat between March 2021 and July 2022. Participants were identified because they had been enrolled either in the PEDFIC2 clinical trial (one child) or in the European compassionate use programme initiated by the manufacturers of odevixibat in July 2021. These corresponded to all children with PFIC associated with myosin 5B deficiency in this programme. The early medical history of Patient 1, enrolled in the PEDFIC2 trial, has been previously reported elsewhere [4].

### Patient follow-up

The children were followed-up for a period of 22 to 39 months according to the centre’s routine practice, which was not standardised. This involved (generally quarterly) visits at which a full clinical evaluation was performed. Blood samples were taken at most, but not all, visits for performing liver function tests. Liver echography was performed when this was judged useful by the physician.

### Data collection

Data was extracted retrospectively from the children’s medical records. Pruritus and sleep disturbances were rated on four-point Likert scales (absent, mild, moderate or severe). The presence or absence of digestive symptoms was noted. Serum levels of bile acids, total bilirubin and alanine aminotransferase (ALT) at each visit were retrieved. Introduction, changes and discontinuation of treatments were documented. Treatments of interest were odevixibat, ursodeoxycholic acid (UDCA) and rifampicin.

## Results

### Patient characteristics

Five children aged 2–10 years, four boys and one girl, were included from four European countries (DE, FR, IT and GB). The characteristics of the patients are presented in Table 1. Biallelic missense variants in *MYO5B* were identified in all patients but one, of which three have not been previously reported. Patient 2 carried a monoallelic non-sense (null) variant in *MYO5B*, inherited from her father, as well as the c.3485G > T/p.(Arg1162Leu) variant of *CFTR* gene of unlikely pathological significance, inherited from the mother. Patient 4 had a history of microvillus inclusion disease and, for this reason, was fed parenterally for the first year of life. He has since been receiving overnight enteral feeding while eating and drinking orally in the day. Patient 1 had a history of repeated episodes of severe acute diarrhoea, which became less frequent and severe after age three years, as previously described [4]. Patient 2 was on the waiting list for a liver transplant at the time of starting odevixibat treatment. In the year before starting odevixibat, all patients presented with moderate to severe pruritus and four out of five had sleep disturbances due to their pruritus, despite treatment with UDCA and rifampicin in all children. At the visit prior to starting odevixibat, total bilirubin levels (median: 62 µmol/L [range: 26–102]) and serum bile acid levels (median: 293 µmol/L [range: 174–380]) were elevated in all children (Table 1). Serum ALT at baseline were slightly elevated in all patients but one, ranging from 37 IU/L (Patient 5) to 100 IU/L (Patient 4).

**Table 1:** Principal patient characteristics prior to odevixibat treatment

	Patient 1	Patient 2	Patient 3	Patient 4	Patient 5
Gender	Boy	Girl	Boy	Boy	Boy
Age at first symptoms^1^	6 months	5 months	7 years	1 month	15 months
Age at diagnosis of myosin 5B deficiency	18 months	15 months	7 years	9 months	25 months
Age at odevixibat initiation	9 years 11 mo	4 years	7 years 2 mo	15 months	3 years
*MYO5B* variants	c.356A > G, p.(Tyr119Cys) homozygous	c.3190 C > T^#^, p.(Arg1064Ter) heterozygous	c.244G > A, p.(Glu82Lys) homozygous	c.1208 C > A^#^, p.(Ala403Asp); c.1361G > A^#^, p.(Cys454Tyr)	c.1072 C > G^#^, p.(Leu358Val); del Exon 1–23
Serum bile acids^2^	293 µmol/L	174 µmol/L	252 µmol/L	380 µmol/L	337 µmol/L
Total bilirubin^2^	54 µmol/L	26 µmol/L	89 µmol/L	102 µmol/L	62 µmol/L
Alanine aminotransferase^2^	41 IU/L	57 IU/L	58 IU/L	100 IU/L	37 IU/L
Prothrombin time/INR^2^	97%	100%	1.0	97%	100%
Pruritus^2^	Moderate	Severe	Severe	Moderate	Severe
Sleep disturbances^2^	Mild	Severe	Severe	Severe	Moderate

^1^First documentation, actual date of first symptoms unknown. ^2^Reported at the visit preceding the start of odevixibat treatment. ^#^*MYO5B* variant not previously reported. Reference values for the laboratory tests are listed in Supplementary Table 1

### Treatment

The initial daily dose of odevixibat ranged from 37.2 to 120 µg/kg (Table 2). This dose was later increased from 37.2 to 40 µg/kg in Patient 3 and from 37.5 to 65 µg/kg in Patient 5, in the latter case because of residual pruritus. In Patients 1 and 4, odevixibat treatment was temporarily interrupted due to diarrhoea. Both boys subsequently resumed the prior dose of odevixibat. In Patient 4, access to treatment was irregular: initially, the daily dose was reduced to 20 µg/kg for three weeks in this patient due to an issue with supply. At the end of this period, the patient experienced an episode of diarrhoea followed by transient paralytic ileus and odevixibat was interrupted for two weeks, before resuming the original daily dose of 40 µg/kg. Subsequently, treatment was interrupted for up to three months when the patient was out of the country, and no treatment has been documented for the last six months of treatment for the same reason. In addition, adherence to treatment in Patients 4 and 5 was suspected to be inadequate. Adherence in Patient 5 improved after a home-care team was provided eight months after starting treatment. Treatment with UDCA and rifampicin was continued under odevixibat at the same dose. These treatments were discontinued after six months of odevixibat in Patient 2 and after two months in Patient 4. Rifampicin but not UDCA was reintroduced in patient 4, to control pruritus which worsened after stopping odevixibat.

**Table 2:** Odevixibat treatment

	Patient 1	Patient 2	Patient 3	Patient 4	Patient 5
Starting daily dose	120 µg/kg	67 µg/kg	37.2 µg/kg	40 µg/kg	37.5 µg/kg
Dose escalation	No	No	48 µg/kg	No	65 µg/kg
Dose at last follow-up	110.3 µg/kg.d) (4800 µg/d)	50 µg/kg.d (1200 µg/d)	40 µg/kg.d (1200 µg/d)	46 µg/kg.d (600 µg/d)	
Duration of follow-up with treatment	39 months	39 months	22 months	22 months	34 months
Treatment interruption	Yes	No	No	Yes	No
Treatment rechallenge	Yes	No	No	Yes, at 20 µg/kg.day then full dose 40 µg/kg.day	No

### Clinical and biological outcomes

**Fig. 1:**
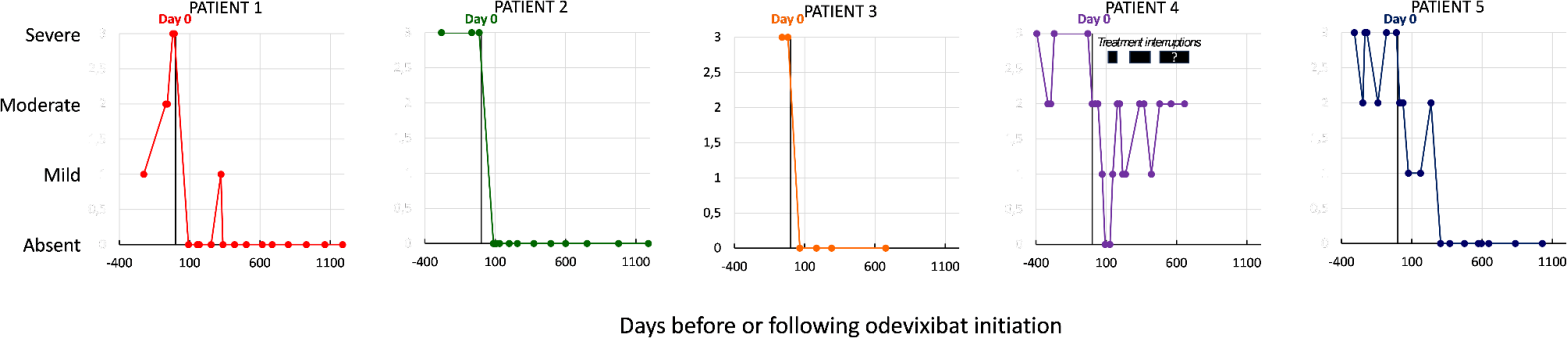
Changes in pruritus score over time

In Patients 1 to 4, pruritus had resolved within three months following treatment initiation (Fig. 1). In general, symptoms remained controlled, with occasional mild pruritus, throughout the on-treatment follow-up period. In Patient 4, moderate pruritus returned when odevixibat treatment was interrupted due to gastroenteritis after UDCA and rifampicin had been stopped. In spite of reintroduction of odevixibat, pruritus persisted and rifampicin was restarted. In Patient 5, mild pruritus resolved completely after a daily dose increase from 37.5 to 65 µg/kg. Sleep disturbances improved in parallel with the resolution of pruritus in all children. Patient 2 has been withdrawn from the waiting list for liver transplantation due to symptomatic remission; her clinical state has remained stable after five years of follow-up.

**Fig. 2:**
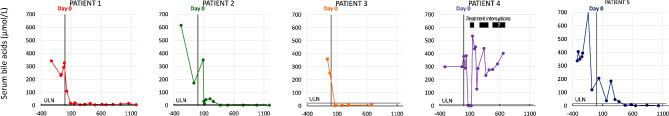
Changes in serum bile acids over time

Serum bile acid levels fell rapidly over the three months following initiation of odevixibat treatment and were close to or within the normal range by six months in four out of five children (Fig. 2). The response was less rapid in Patient 5, who was poorly compliant during the first months of treatment. On-treatment sBA levels remained within or close to the normal range throughout the follow-up (except in Patient 4), which now extends to around three years in three patients. In Patient 4, treatment was interrupted after four months due to a gastrointestinal infection and a transient episode of paralytic ileus, when sBA levels rapidly returned to above pre-treatment levels. Once treatment with odevixibat (but not UDCA) was reinstated, sBA levels remained elevated for the rest of the follow-up, although it should be noted that UDCA was never reintroduced. In Patient 1, odevixibat was also interrupted for one week due to diarrhoea, but sBA levels were not measured during or immediately after this event.

**Fig. 3:**
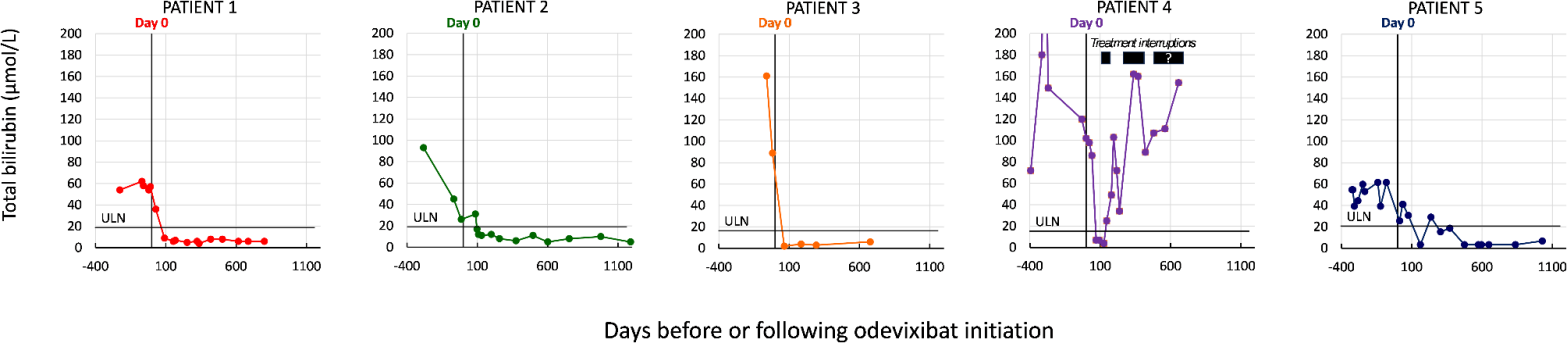
Changes in total bilirubin over time

In parallel, total bilirubin levels in all children descended into the normal range, within three months of treatment initiation (Fig. 3). Patient 4 experienced a progressive and persistent increase in bilirubin following interruption of all treatments. Despite reintroduction of odevixibat, bilirubin levels never normalised again. The other children all maintained normal bilirubin levels throughout the follow-up period.

### Safety

Overall, the digestive tolerability of odevixibat was good. Patient 1 presented one episode of diarrhoea, which required hospitalization, and rehydration during which odevixibat treatment was interrupted for one week. Apart from this serious episode, this patient presented episodic digestive symptoms similar to those occurring before odevixibat treatment. Nonetheless, these symptoms were in fact less frequent and less severe than before treatment and no aggravation was observed whilst on odevixibat. Patient 4 experienced an episode of diarrhoea due to enteropathogenic *E. coli* followed by transient paralytic ileus. Although these manifestations were not deemed to be related to treatment, odevixibat was nevertheless interrupted for two weeks, before resuming the original daily dose of 40 µg/kg. Diarrhoea did not recur following resumption of odevixibat treatment in this patient. No new or worsening gastrointestinal symptoms were observed in the other children. No adverse events were documented that were considered potentially related to odevixibat.

**Fig. 4:**
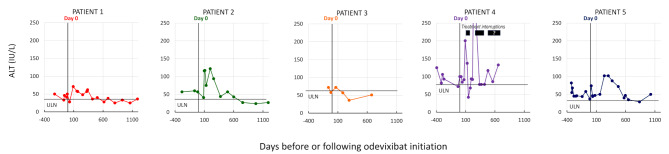
Changes in alanine aminotransferase over time

Alanine aminotransferase levels remained unchanged in Patients 1 and 3 after starting odevixibat. In Patients 2, 4 and 5, a transient rise in ALT was observed between three and twelve months after treatment initiation (Fig. 4). In all patients except Patient 4, ALT levels were below or close to the upper limit of normal at the last follow-up.

## Discussion

Our observations in this case series indicate that treatment with odevixibat reduced levels of sBA and bilirubin in children with PFIC associated with myosin 5B deficiency; levels normalised within 3 months in all patients except Patient 5. Improvement in pruritus and sleep was also seen following odevixibat treatment. The only child on the waiting list for liver transplantation before starting odevixibat was removed from the list due to symptomatic remission (Patient 2). The outstanding clinical and biological benefits were sustained for as long as the children were followed (up to 40 months), except Patient 4 who relapsed following interruption of treatment and failed to respond to rechallenge.

The methodology of this open-label retrospective study does not permit assignment of causality to the clinical benefits observed. While pruritus is a subjective symptom and can be prone to placebo effects [22], biological markers including sBA and total bilirubin are objective markers which are not subject to placebo effects. The time course of changes in symptoms and biomarkers is also consistent with a real treatment effect, with rapid improvement in all outcome measures after treatment onset. In all patients but Patient 4, these benefits persisted for at least two years. Taken together, these arguments suggest a real treatment effect of odevixibat in our patients.

All patients previously received rifampicin or UDCA prior to initiation of odevixibat and initially continued to receive these treatments, which could confound interpretation of these data. Importantly, in two children UDCA and rifampicin treatments were subsequently interrupted without any recurrence of pruritus or perturbation of liver biology, including sBA. This suggests that, in some children with PFIC, IBAT inhibition has the potential to control cholestasis completely, even when used as a monotherapy. In contrast, Patient 4 did not respond to odevixibat alone after treatment interruption and having responded previously to a combination of odevixibat and UDCA. Although other factors could have contributed to the lack of response to odevixibat monotherapy in this patient, a synergistic effect between UDCA and odevixibat cannot be excluded in certain patients.Further studies may help to clarify this issue.

In our patients, the initial daily dose of odevixibat ranged from 37.2 to 120 µg/kg, with two patients receiving dose increases. This is in line with the recommended dose of odevixibat of 40 µg/kg administered orally once daily in the morning. It is also suggested that improvement in pruritus and reduction of sBA may occur gradually in some patients after initiating odevixibat, and if an adequate clinical response is not achieved after 3 months of continuous therapy, the dose may be increased to 120 µg/kg/day.

The benefit of IBAT inhibitors in PFIC has also recently been demonstrated in a randomized placebo control study of another drug of this class, maralixibat (MARCH-PFIC), which included four patients PFIC associated with *MYOB5* variants, two in each group [23]. In this study, treatment with maralixibat was shown to improve pruritus and decrease sBA level in all forms of PFIC studied, including in those with myosin 5B deficiency. Importantly, in the single myosin 5B deficient patient who had elevated bilirubin at baseline and was treated with maralixibat, bilirubin normalised on treatment [23]. This finding is entirely consistent with the changes reported in the present study with odevixibat.

The precise mechanism responsible for the specific PFIC phenotype in myosin 5B deficiency has not been fully elucidated [1, 7]. The observation of reduced bile acid levels in bile in one patient with myosin 5B deficiency-associated PFIC [4] suggests a secondary bile acid transport defect, perhaps involving impaired BSEP trafficking [10]. However, the reduction of biliary BA in this child was modest, especially compared to the reduction observed in PFIC2 [24]. Patients with PFIC associated with myosin 5B deficiency generally exhibit a milder liver disease than those with PFIC2 [12, 25]. Moreover, a genotype-phenotype correlation has been suggested in myosin 5B deficient patients, with patients carrying biallelic missense variants having milder forms of cholestasis than those carrying null variants [26]. The patients reported here all carried biallelic missense variants except Patient 2 who carried a single monoallelic null variant and Patient 5 who harboured a monoallelic missense variant and a deletion in the other allele. These considerations suggest that the biliary BA transport defect in PFIC associated with myosin 5B deficiency may be limited, especially in those carrying biallelic missense variants, and therefore amenable to treatments that prevent reabsorption of BA. Surgical partial external biliary diversion has indeed been reported to be effective in certain patients with PFIC associated myosin 5B deficiency [4, 7, 11, 16]). Odevixibat and maralixibat provide chemical biliary diversion by inhibition of IBAT and were highly effective, further supporting this set of hypotheses.

In two patients (Patients 4 and 5) symptoms and biomarkers fluctuated over time and treatment response was not reproducible in Patient 4. This could be due to issues with access to treatment and inadequate adherence. Both these children came from immigrant families who experienced difficulties or delays in getting residence status and local health insurance, and this may have compromised their ability to access regular medical follow-up and medication delivery. Given the mechanism of action of odevixibat, which is a reversible inhibitor of the IBAT [27] with minimal systemic absorption, continuous treatment is important and the present results show that drug interruption results in rapid symptom recurrence. For treating physicians, it is important to emphasise to patients’ families the importance of good adherence, all the more so since these treatments are currently very expensive.

Two patients experienced GI events, with one patient experiencing an episode of severe diarrhea requiring hospitalization. Despite this event, this patient experienced less frequent and less severe episodic digestive symptoms than prior to odevixibat initiation. These findings are consistent with the safety profile of odevixibat reported in the Phase III PEDFIC clinical trial program, during which GI events reported with odevixibat were generally mild-to-moderate [20, 21]. The absence of any new tolerability or safety signals with odevixibat is reassuring with respect to the requirement for long-term treatment with odevixibat. It is pertinent to emphasise the good digestive safety and tolerability of this medication observed here, given the fact that myosin 5b deficiency may be responsible for a spectrum of digestive manifestations. These could theoretically worsen upon treatment with IBAT inhibitors due to their mechanism of action. No such worsening was observed in the study, with one patient presenting less digestive manifestations on treatment than he did before.

The principal limitations of this study, besides its retrospective nature, were the small number of patients included and the lack of a formalised follow-up. Pruritus was evaluated by a four-point Likert scale (commonly used in practice settings) rather than using a validated pruritus tool (such as those more often utilised in clinical trials). Even though the children studied in this report were all treated and followed in routine clinical practice rather than in a controlled clinical trial, our findings provide useful information on the effectiveness and safety of IBAT inhibitors in patients with myosin 5b deficiency. Given the very low incidence of the disease on the one hand, and the fact that these medications are already licensed for the treatment of PFIC on the other, it is very unlikely that new controlled studies will be performed. Further data will come from real world studies and potentially from patient registries [25]. The present study demonstrates the feasibility and the interest of performing such studies.

In conclusion, this case series indicates that treatment with odevixibat is effective and safe in children with PFIC associated with myosin 5B deficiency and encourages further research into the utility of this medication in rare forms of PFIC.

## Electronic supplementary material

Below is the link to the electronic supplementary material.

Supplementary Table 1. Reference normal values for laboratory tests

## Data Availability

Readers who are interested in further information on the study may contact the authors.
