# Supplementary material for: Odevixibat therapy in progressive familial intrahepatic cholestasis with MYO5B variants: a retrospective case series
Source: Orphanet J Rare Dis. 2025 May 12;20:227. doi: 10.1186/s13023-025-03728-x (PMC12070763; doi:10.1186/s13023-025-03728-x)
Supplement: Supplementary file 1 — Supplementary Table 1. Reference normal values for laboratory tests [file 13023_2025_3728_MOESM1_ESM.docx]

# SUPPLEMENTARY MATERIAL

Supplementary Table 1. Reference normal values for laboratory tests

| Serum bile acids | <10 µmol/L | Patients 1, 2 and 5 |
| --- | --- | --- |
|  | <14 µmol/L | Patient 3 |
|  | <8 µmol/L | Patient 4 |
| Total bilirubin | <17 µmol/L | Patients 1, 2, 4 and 5 |
|  | <20 µmol/L | Patient 3 |
| Alanine aminotransferase | <35 IU/L  <40 IU/L | Patient 1  Patients 2 and 5 |
|  | <55 IU/L | Patient 3 |
|  | <78 IU/L | Patient 4 |
| Prothrombin time | >70% | Patient 1,2, 4 and 5 |
| INR | 0.8–1.2 | Patient 3 |
